# Effects of High-Intensity Anaerobic Exercise on the Scavenging Activity of Various Reactive Oxygen Species and Free Radicals in Athletes

**DOI:** 10.3390/nu15010222

**Published:** 2023-01-01

**Authors:** Yuri Sawada, Hiroshi Ichikawa, Naoyuki Ebine, Yukiko Minamiyama, Ahad Abdulkarim D. Alharbi, Noriaki Iwamoto, Yoshiyuki Fukuoka

**Affiliations:** 1 Graduate School of Medical Life System, Faculty of Life and Medical Science, Doshisha University, Kyoto 610-0394, Japan; 2 Graduate School of Health and Sports Science, Doshisha University, Kyoto 610-0394, Japan; 3 Food Hygiene and Environmental Health Division, Graduate School of Life and Environmental Sciences, Kyoto Prefectural University, Kyoto 606-8522, Japan

**Keywords:** Wingate exercise test, reactive oxygen species, athlete, high-intensity exercise

## Abstract

High-intensity exercise in athletes results in mainly the production of excess reactive oxygen species (ROS) in skeletal muscle, and thus athletes should maintain greater ROS scavenging activity in the body. We investigated the changes in six different ROS-scavenging activities in athletes following high-intensity anaerobic exercise. A 30-s Wingate exercise test as a form of high-intensity anaerobic exercise was completed by 10 male university track and field team members. Blood samples were collected before and after the exercise, and the ROS-scavenging activities (OH•, O_2_•^−^, ^1^O_2_, RO• and ROO•, and CH_3_•) were evaluated by the electron spin resonance (ESR) spin-trapping method. The anaerobic exercise significantly increased RO• and ROO• scavenging activities, and the total area of the radar chart in the ROS-scavenging activities increased 178% from that in pre-exercise. A significant correlation between the mean power of the anaerobic exercise and the ^1^O_2_ scavenging activity was revealed (r = 0.72, *p* < 0.05). The increase ratio in OH• scavenging activity after high-intensity exercise was significantly greater in the higher mean-power group compared to the lower mean-power group (*n* = 5, each). These results suggest that (*i*) the scavenging activities of some ROS are increased immediately after high-intensity anaerobic exercise, and (*ii*) an individual’s OH• scavenging activity responsiveness may be related to his anaerobic exercise performance. In addition, greater pre-exercise ^1^O_2_ scavenging activity might lead to the generation of higher mean power in high-intensity anaerobic exercise.

## 1. Introduction

Although prolonged submaximal aerobic exercise has been demonstrated to induce oxidative stress [1,2,3,4], there are few data on the effects of short-term supramaximal anaerobic exercise, especially in humans. During exercise, ischemia-reperfusion produces reactive oxygen species and free radicals in the vessels of skeletal muscle [5]. Supramaximal anaerobic exercise has been associated with a substantial degree of lactic acidosis in both blood and muscle [6] and with a major increase in plasma catecholamine levels [7]. Such exercise also stimulates the catabolism of purines to xanthine and urate, as evidenced by plasma urate accumulation [8]. However, if the reactive oxygen species (ROS) and free radicals produced during or by the high-intensity exercise are not properly scavenged, skeletal muscle cells are oxidized, resulting in poor exercise performance as well as post-exercise muscle damage and the potential development of various diseases [9].

The results of the only study that we could find that used the Wingate exercise test demonstrated that the paradoxical decrease in plasma thiobarbituric acid reactive substances (TBARS) was correlated with the peak power developed during the Wingate exercise test (r  =  −0.7) [10]. That observation suggested that the decreased TBARS level is not a suitable marker during the Wingate exercise test. The observation of a decrease in erythrocyte glutathione (GSH) might be explained by the consumption of GSH to scavenge free radicals such as superoxide anion and singlet oxygen [11]. It was also reported that singlet oxygen activated mitochondrial respiration by promoting the activity of cytochrome *C* oxidase [12,13] and complex VI of the mitochondrial electrical transport chain stimulated the production of ATP [14]. Since performing the Wingate exercise test would induce a significant increase in lipid radical production and mitochondrial bioenergetics [15], we have speculated that athletes need to maintain greater ROS and free radical scavenging activity against excessive oxidative stress after they engage in a short period of high-intensity anaerobic exercise such as the Wingate exercise test.

The electron spin resonance (ESR) spin-trapping method detects ROS and free radicals by reacting unstable and short-lived ROS and free radicals with various spin-trapping agents to convert them into spin adducts, which are relatively stable and long-lived substances [16]. Since the ESR method generates ROS and free radicals by light irradiation, the sample of interest is always able to produce a constant amount of reactive oxygen and free radicals without affecting the radical production system. ESR also accurately measures the elimination activity separately; for example, the activity of the six reactive oxygen free radicals, i.e., hydroxyl radical (OH•), superoxide radical (O_2_•^−^), alkyloxyl radical (RO•), alkyl peroxyl radical (ROO•), methyl radical (CH_3_•), and singlet oxygen (^1^O_2_) by using the multiple free-radical scavenging (MULTIS) method [17]. A study using ESR demonstrated that contracting skeletal muscles produce ROS and vitamin E deficiency exacerbates the production of ROS in both liver and muscle [18], revealing the important role of vitamin E in protecting biological membranes from exercise-induced oxidative damage.

We chose the Wingate exercise test for the present investigation because it strongly stimulates both the adenosine triphosphate-phosphocreatine (ATP-PCr) system and the glycolytic system [19] and thus activates purine catabolism [20] and the production of lactic acid [21]. During a prolonged, electrically stimulated leg exercise, oxidants contributed to muscle fatigue [22,23], and athletes have often shown increased total antioxidant capacity in response to the oxidative stress imposed by intense physical activity [24].

Accordingly, we addressed two specific questions in this study: (*i*) whether abrupt high-intensity anaerobic exercise causes muscle fatigue with greater lactate accumulation and generates a larger amount of ROS simultaneously; and (*ii*) whether the singlet oxygen in ROS associated with mitochondrial respiration is related to exercise performance. To examine these questions, we investigated the relationship between serum ROS and free radical scavenging activity and athletes’ exercise performance in order to clarify the process of changes in several ROS and free radical scavenging activities in vivo during abrupt high-intensity anaerobic exercise.

## 2. Materials and Methods

### 2.1. Participants

The study subjects were 10 male members of a university athletic association team (age 21  ±  1 years, height 170.9  ±  4.6 cm, weight 65.6  ±  2.7 kg, body mass index [BMI] 22.5  ±  0.9, body fat percentage 10.9  ±  1.5%; mean  ±  standard deviation [SD]). All subjects had been training for track and field short-distance events for ≥2 years. The physical training status of the subjects depended on their individual sports activities. They had been consistently training in their sports 4–5 days/week and usually had sport matches or a day off on weekends.

Since tobacco and alcohol are environmental factors that produce ROS and free radicals, the subjects were non-smokers, and the prohibition of alcohol consumption on the day before their study participation was specified [25]. Since caffeine has been reported to improve exercise performance [26], the consumption of foods high in caffeine was also banned. To avoid sleep deprivation, the subjects were instructed to finish eating by 10:00 pm on the day before the study and to get ≥6 hr of sleep. This study was conducted with the approval of the Ethical Review Committee for Human Subjects Research at Doshisha University (19051).

### 2.2. Exercise Protocol

After a subject came to the laboratory, he was seated in a chair and remained in a resting state for 30 min. Next, as a warm-up, he pedaled a bicycle ergometer (Aerobike 75XL III; Konami, Tokyo, Japan) at 60 watts and 50 rpm for 5 min, followed by an approx. 5 min rest. The subject then continued pedaling in order to perform the Wingate exercise test with an interval of about 30 s to move another ergometer. The Wingate exercise test is a typical estimation of anaerobic power that uses another bicycle ergometer (Powermax VII; Konami, Tokyo), and it was performed in this study with the Powermax VII as a cycling test in which the pedaling was driven at 7.5% of the subject’s body weight for 30 s under maximum effort [10,27].

The rotation speed is calculated using the number of revolutions of the pedals, and the power is calculated from the distance of one rotation of the pedals and the load (kp) of the Powermax VII. It is possible to calculate the mean power for 30 s [28]. During the Wingate exercise test, the subject sits in the saddle of the bicycle ergometer with his knees lightly bent at the lowest point of the pedals, so that the subject could pedal with full force and assume the optimal position by considering his subjective sensations. Throughout the exercise and recovery, the subject’s heart rate (HR) was measured with an HR monitor (A370, Polar, Finland).

The cool-down period of light-intensity exercise during the recovery period after strenuous exercise has the advantage of controlling a subject’s post-exercise behavior, and thus in the present protocol the subject was moved to first bicycle ergometer (Aerobike 75XL III; Konami, Tokyo) immediately following the Wingate exercise test, and he performed cycling at the light work rate of 40 watts and 50 rpm. After this active recovery, the subject was then seated in a chair and remained at rest for 10 min.

For the assessment of exercise-induced changes in serum ROS and free radicals, blood samples were collected from each subject at rest before the warm-up and at the end of the cooling-down period. The subject’s blood lactate concentration ([Lac]) was determined once when he was at rest before warming up and four times after the completion of the Wingate exercise test. All tests were completed in a temperature-controlled laboratory (22  ±  0.1 °C, relative humidity 50  ±  0.8%).

### 2.3. Measurements

Prior to the exercise test, the subject’s height, weight, BMI, and body fat percentage were measured. A digital audiometer (DNS-90, Muratec-KDS, Tokyo) was used to measure height, and an impedance-based body composition analyzer (Inner Scan V, BC-612, Tanita, Tokyo, Japan) was used to measure weight and body fat percentage. The measured parameters were the mean power, peak power, peak rotation speed, and peak time to peak obtained from the Powermax VII, as well as HR, [Lac], and ROS and free radical scavenging activity in the serum.

A 5-μL blood sample was collected from the subject’s fingertip with a blood paracentesis device (Acelet II, Sanwa Kagaku Kenkyusho, Tokyo, Japan), and the blood was quickly analyzed by a simple blood lactate analyzer (Lactate Pro 2, Arkray, Kyoto, Japan). The peak value of the five samples (before the warm-up and four times after the exercise test) was defined as the peak [Lac]. It was observed that the peak [Lac] values occurred at ~3–5 min after high-intensity exercise [29], and we thus set the blood sampling time-points as before (i.e., rest) and during recovery at 1, 3, 5, and 15 min after the completion of the Wingate exercise test.

### 2.4. Human Serum

The pre- and post-exercise blood samples collected from the 10 subjects were placed in a vacuum collection tube (Benoject II, Terumo, Tokyo, Japan) and stored on ice. After the exercise test, the samples were centrifuged in a centrifuge (CT15RE, Hitachi, Tokyo, Japan) at 1630× *g* for 6 min at 4 °C, and the supernatant was collected and stored at −80 °C.

### 2.5. Measurement of the Plasma Free Radical Species Scavenging Capacity

The plasma scavenging capacity for the different free radical species was measured according to the MULTIS method reported by Oowada et al. [17]. and Kobayashi et al. [30]. Spin trapping agents, i.e., 5-(2,2-dimethyl-1,3-propoxy cyclophosphory)-5-methyl-1-pyrroline *N*-oxide (CYPMPO, Radical Research, Tokyo) and *N*,*N*,*N*′,*N*′-tetramethyl-p-phenylenediamine (TMPD, Radical Research, Tokyo) were used to measure the scavenging capacity of each free radical with an X-band Microwave Unit ESR (RE Series, Jeol, Tokyo). WIN-RAD software (ver. 1.30, Radical Research, Tokyo) was used for the data analyses.

Briefly, we prepared a solution containing 20 µL of 1:5 diluted plasma samples, 20 µL of 100 mM CYPMPO, 20 µL of 10 mM dimethylene triamine pentaacetic acid (DTPA, Wako, Osaka, Japan), 20 µL of distilled water, and 20 µL of 100 mM H_2_O_2_ for hydroxy radical (OH•) generation. This solution was exposed to UV light (Supercure-203, San-Ei Electric, Osaka) for 5 s (total reflection mirror, hot-cut filter). CYPMPO was used as a spin trapping reagent to capture any radicals produced in the solution. The height of the fifth signal of each sample was recorded. The fifth signal of control was set to 100% and compared with each sample to obtain a relative ratio.

The standard calibration curve was determined using glutathione disulfide (GSSG, Wako). The relative ratios of the control and each concentration were set as I0 and I, respectively, when GSSG was added to each sample. In the graph created from these results, the *y*-axis and *x*-axis represented I0/I-1 and the concentration of GSSG (mM), respectively. For other free radicals, i.e., superoxide radical (O_2_•^−^), alkoxy radical (RO•), methyl radical (CH_3_•), alkylperoxyl radical (ROO•), and singlet oxygen (^1^O_2_), each suitable reaction mixture was used and measured for each condition. When the singlet oxygen scavenging activity was measured, TMPD was used as a spin trap agent instead of CYPMPO. The typical spectrometer settings were as follows: field modulation width, 0.1 mT; microwave power, 6 mW; field scan width and rate, ±7.5 mT/2 min; and time constant, 0.1 s.

### 2.6. Statistical Analyses

The various reactive oxygen and free radical scavenging activities were analyzed with SPSS software (ver. 25, IBM Japan, Tokyo, Japan) and are presented as the mean ± standard deviation (SD). The distribution of the data was assessed using the Shapiro–Wilk test, and none of the data were disturbed. Therefore, the parametric tests were applied, and paired t-tests were performed to compare the scavenging activities of the reactive oxygen and free radical species at pre and post the Wingate exercise test, and also was performed for the up-rate data of OH• scavenging activity from pre to post, which we divided into two groups: a higher mean-power group (*n* = 5) and a lower mean-power group (*n* = 5). Since the subjects were members of a university athletic association team and there was a significant correlation between mean power of the Wingate test and 100m sprint record [31], we simply divided into two groups. The effect size (ES) was calculated as Cohen’s d between the trials’ change from pre- to post-exercise to elucidate the practical significance of sever anaerobic exercise. The criteria to interpret the magnitude were as follows: ≥0.20 (small), ≥0.50 (medium), and ≥0.80 (large) [32], A correlation analysis was performed for the mean power in the Wingate exercise test and the pre-exercise ^1^O_2_ scavenging activity and peak [Lac] values by determining the Pearson’s correlation coefficient. Pearson’s correlation coefficient (r) for liner relationship graph was also calculated and can be interpreted as: ≥0.20 (weak), ≥0.40 (moderate), ≥0.70 (strong), ≥0.90 (very strong) [33,34]. The significance level was set at 5%.

## 3. Results

### 3.1. Wingate Anaerobic Test

For all 10 subjects, the mean power was 583  ±  15 watts and the peak power was 742  ±  29 watts. The number of peak cycling repetitions was 153  ±  5 rpm to reach the mean time of 6.4  ±  0.5 s. The peak HR value during the 30-s Wingate exercise test was reached at 176  ±  3 bpm, thus, the mostly exhaustion in the subjects would be performed.

### 3.2. Blood Lactate Concentration

The blood lactate concentration [Lac] was 1.9  ±  0.7 mM at rest before the Wingate exercise test, and it appeared to reach the peak value of 18.0  ±  3.4 mM at 3 min after the completion of the Wingate exercise test (at 1 min recovery, 14.0  ±  5.3 mM; at 5 min recovery, 16.3  ±  4.3 mM; and at 15 min recovery, 11.2  ±  2.6 mM). There was a significant correlation between peak [Lac] and the mean power of the Wingate exercise test (r = 0.838, *p* = 0.002), indicating that mostly glycolic metabolism contributed to the metabolic activation during the Wingate exercise test [35].

### 3.3. Measurement of Reactive Oxygen and Free Radical Scavenging Activity

**Figure 1:**
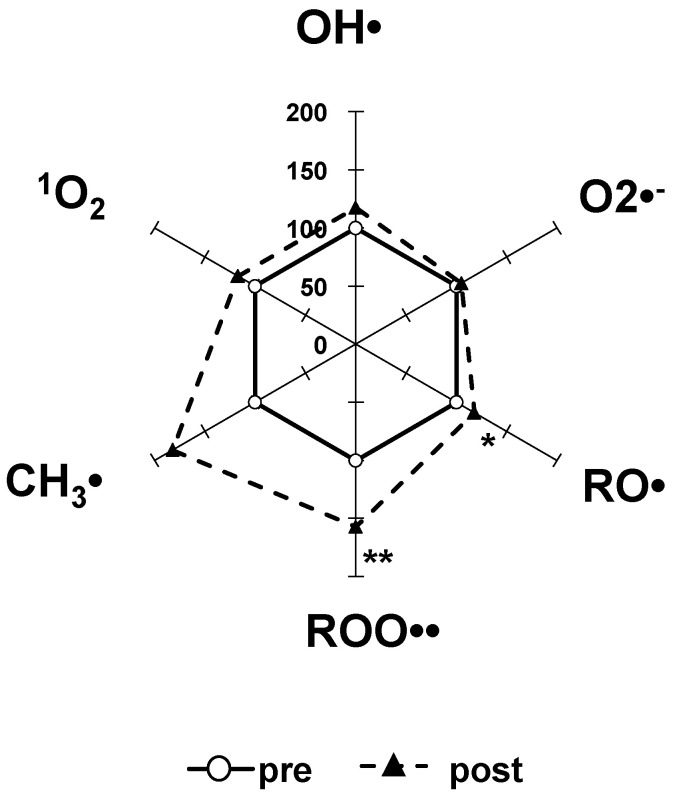
The changes in the scavenging activity of six reactive oxygen species in the 10 subjects’ serum (%) from pre-exercise (100%) to post-exercise. The standard substance equivalent of each mean pre-exercise value was set as 100%, and the relative post-exercise values were calculated. The total area of the radar chart increased 178% from that in pre-exercise. * *p* < 0.05, ** *p* < 0.01 vs. pre-exercise.

We corrected the relative post-exercise values of all of the ROS free radical scavenging activities by using the pre-exercise value as the standard equivalent of 100% [17]. The area ratio of the radar chart was 178% for post-exercise compared to 100% for pre-exercise (Figure 1).

**Figure 2:**
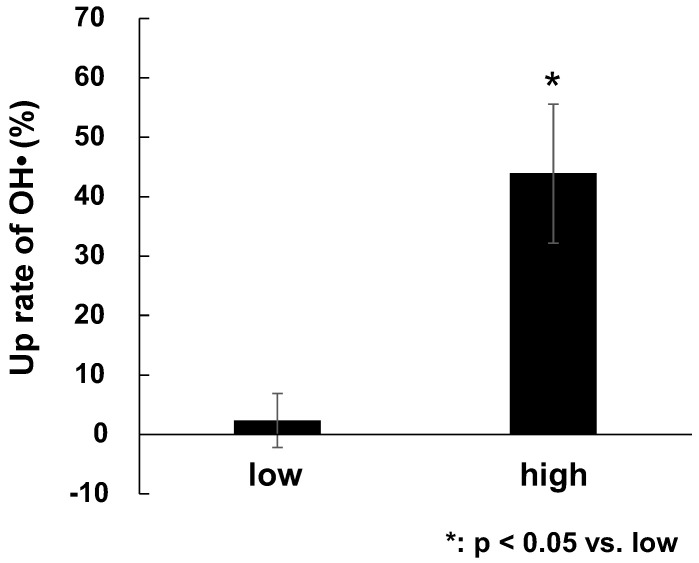
The rate of increase in the hydroxyl-radical scavenging activity in the low- and high-mean power groups in the Wingate exercise test. The group with high-mean power exhibited significantly greater •OH scavenging activity after the high-intensity exercise (*p* = 0.018).

The mean power obtained from the Wingate exercise test was used in a subgroup analysis of the high- (*n* = 5) and low-mean power (*n* = 5) groups. The rate of increase in OH• scavenging activity was thus assessed in the group with high-mean power and the group with low-mean power in the Wingate exercise test. Although the post-exercise OH• scavenging activity was 17.5% higher than that at pre-exercise, the OH• scavenging activity converted by using the GSH concentration was not significantly increased compared to the post-exercise activity (pre: 97.8  ±  14.7 mM, post: 115.0  ±  14.8 mM, *p* = 0.053, ES = 0.35). The increase rate of OH• scavenging activity was 43.9  ±  11.7% in the high-mean power group, which is significantly greater than the 2.4  ± 4.5% in the low-mean power group (Figure 2, *p* = 0.018).

The RO• scavenging activity increased by 17.6% post-exercise, and the RO• scavenging activity converted by using the Trolox concentration was significantly augmented from pre- (6.9  ±  1.3 mM) to post-exercise (8.1  ±  1.4 mM, *p* = 0.04). The ROO• scavenging activity also increased by 57.1% post-exercise; the ROO• scavenging activity converted by α-lipoic acid concentration revealed that the pre-exercise value was 5.4  ±  1.0 mM and the post-exercise value was equivalent to 8.5  ±  1.1 mM, a significant increase (*p* < 0.001).

Even though the CH_3_• scavenging activity was increased 81.9% post-exercise, the analyses demonstrated no significant CH_3_• scavenging activity change when converted by using the bisphenol A concentration (pre: 28.5  ±  10.5 mM, post: 51.8  ±  17.9 mM, *p* = 0.072, ES = 0.47). Similarly, the O_2_•^−^ scavenging activity converted using the superoxide dismutase concentration was not significantly increased following the Wingate exercise test (pre: 6.9  ±  1.9 mM, post: 7.3  ±  1.9 mM, *p* = 0.674, ES = 0.06).

### 3.4. The Relationship between ^1^O_2_ Scavenging Activity and the Mean Power in the Wingate Exercise Test

**Figure 3:**
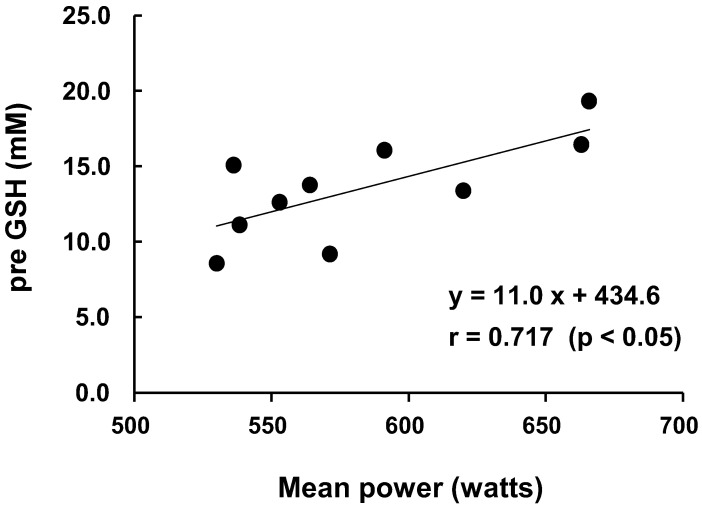
The relationship between mean power and singlet oxygen scavenging activity pre-exercise. A significant correlation between the mean power and singlet oxygen scavenging activity before exercise was observed (r = 0.717, *p* = 0.02).

The ^1^O_2_ scavenging activity post-exercise was increased by 17.2% compared to pre-exercise, but the ^1^O_2_ scavenging activity converted using the GSH concentration was not significantly increased (pre: 13.6  ±  1.0 mM, post: 15.9  ±  1.1 mM, *p* = 0.127, ES = 0.66). However, the ^1^O_2_ scavenging activity in high-mean power group tended to be higher than in low-mean power group (ES = 0.83). Interestingly, there was a significant positive correlation between the mean power and the pre-exercise ^1^O_2_ scavenging activity (r = 0.717, *p* = 0.02) (Figure 3).

## 4. Discussion

To the best of our knowledge, this is first study to investigate the effects of severe anaerobic exercise on the scavenging activity of various ROS and free radicals in the body by the MULTIS method using human serum. The results of our analyses revealed that high-intensity anaerobic exercise induced a significant increase in the scavenging activities of RO• and ROO• and the total area of the radar chart increased 178% from that in pre-exercise (Figure 1). A tendency for an increased in the scavenging activities of OH•, ^1^O_2_, and CH_3_• was also observed (Figure 1).

It has been reported that the oxygen uptake of the body during heavy exercise is 10–15 times greater than that at rest, and the oxygen uptake by working muscle is roughly 100 times greater [36]. We and others have speculated that the production of ROS and free radicals in the body should therefore be increased, resulting in tissue damage, inflammation, and various oxidative disorders in the body [18,37,38]. Our present findings emphasized that severe anaerobic exercise rapidly increased the scavenging activities of some serum ROS and free radicals. We also observed that the changes in these scavenging activities are remarkably different for each ROS and free radical in response to high-intensity exercise stress.

In a study of antioxidant capacity evaluated after exercise, it was observed that the antioxidant capacity increased after the exercise and was further increased at 10 min after the exercise [39]. This antioxidant phenomenon might be due to the release of antioxidant-related myokines from skeletal muscle into the bloodstream. The release of myokines from skeletal muscle cells by physical stimulation has been confirmed in vitro [40,41,42,43,44], but a more detailed relationship between antioxidants and myokines has not been discussed. Our present results suggest the possibility that the abrupt release of antioxidant myokines took place within a short period of the Wingate exercise test (i.e., 30 s). The amount of myokines released into the bloodstream is thought to depend on the amount of myokines stored in the skeletal muscle [45]. However, a clear mechanism explaining the rapid increase in ROS and free radical scavenging activity during high-intensity exercise has not been identified, and the elucidation of this mechanism is a major challenge for future research, since our present investigation revealed that cells also exhibit antioxidant defensive responses to rapid oxidative stress exposure, such as that provided by anaerobic exercise.

We observed that the RO• and ROO• scavenging activities were significantly increased by the anaerobic exercise. Lipid radicals were reported to be significantly increased after 20 min of the Wingate test [10,46]. They reported that acute exhaustive exercise increased the concentration of oxidized high-density lipoprotein (HDL) lipid, which indicates that HDL has an active role in the removal of lipid peroxides. Thus, the rapid production of lipid radicals in the body may be responsible for the increased scavenging activity of the lipid radicals as a way to protect the body [10].

The present study also obtained a new finding, i.e., the significant positive correlation between the mean power in the Wingate exercise test and the pre-exercise singlet oxygen scavenging activity (Figure 3). Mitochondria are the dominant site of ROS production in skeletal muscles [47,48], but NADPH oxidase is likely to play a key role in the contraction-induced production of ROS [49]. Although there are few publications about the significance of singlet oxygen scavenging activity, it was reported that astaxanthin, which is believed to scavenge singlet oxygen [50], may act on mouse mitochondria during exercise to enhance their athletic performance [51].

There was a significant positive correlation between the mean power and the pre-exercise ^1^O_2_ scavenging activity (Figure 3). It was recently shown that laser irradiation can produce singlet oxygen without photosensitizers, leading to increases in the mitochondrial membrane potential and the rate of mitochondrial respiration [14]. Singlet oxygen activated mitochondrial respiration by promoting the activity of cytochrome *C* oxidase [12,13] and/or complex VI of the mitochondrial electrical transport chain, and it stimulated the production of ATP [52]. Since increasing the singlet oxygen scavenging activity of the skeletal muscle prior to exercise is protective against the rapid oxidative stress caused by exercise, it would make sense for athletes to correlate pre-exercise singlet oxygen scavenging activity with athletic performance. However, it is not clear what mechanisms underlie the association between human athletic performance and the pre-exercise singlet oxygen scavenging activity. Further investigations of this association are necessary.

Regarding the relationship between exercise performance and the scavenging activity of ROS and free radicals other than singlet oxygen, no significant increase in OH• scavenging activity was observed in this study. However, when we compared the high and low mean-power groups we observed that the rate of increase in OH• scavenging activity was significantly higher in the group with high mean power (Figure 2, *p* < 0.05). This may indicate that the amount of antioxidant myokines released immediately after exercise varies among individuals [44]. In other words, exercise performance may be correlated with how much OH•-scavenging myokines an athlete can release. Our results indicate that subjects with higher exercise performance are more responsive to the oxidative stress caused by anaerobic exercise interventions, suggesting that those who can respond quickly to rapid oxidative stress caused by high-intensity exercise are capable of achieving higher performance [15].

As the subjects of the present study were limited to athletes, the effects on subjects without exercise habits are unknown. In addition, since only anaerobic exercise was performed in this study, it is unclear whether aerobic exercise is specific to various ROS and free radical scavenging activities. We plan to evaluate exercise performance by providing the subjects with functional foods or a physical therapy intervention that alters antioxidant capacity and to examine the effects on the scavenging activities of various ROS and free radicals.

## 5. Conclusions

Our study using the Wingate test performed by university-level athletes demonstrated that (*i*) the scavenging activity of some ROS and free radicals in serum are increased immediately after this high-intensity anaerobic exercise and (*ii*) the responsiveness of OH• scavenging activity may be related to exercise performance in trained athletes, who counteract the ROS generated by daily training. Our findings also suggest that greater pre-exercise ^1^O_2_ scavenging activity might lead to the generation of higher mean power in a high-intensity anaerobic exercise test by inhibiting the mitochondrial dysfunction that occurs in response to oxidative stress.

## Data Availability

The datasets generated and/or analyzed during the current study are available from the corresponding author on reasonable request.
